# Using preprocessed datasets to construct and interpret multiclass identification models

**DOI:** 10.3389/fpls.2025.1597673

**Published:** 2025-08-20

**Authors:** Cong Wang, Yufeng Fu, Ran Wan, Le Zhao, Hongbo Wang, Junwei Guo, Qiang Liu, Shan Li, Shengtao Ma, Zhicai Wang, Wei Huang, Huimin Liu, Song Yang, Cong Nie

**Affiliations:** ^1^ Key Laboratory of Tobacco Chemistry, Zhengzhou Tobacco Research Institute of China National Tobacco Corporation (CNTC), Zhengzhou, China; ^2^ Technology Center, China Tobacco Henan Industrial Co., Ltd., Zhengzhou, China; ^3^ Technology Center, China Tobacco Gansu Industrial Co., Ltd., Lanzhou, China

**Keywords:** multiclass identification, preprocessed data, kernel support vector machine, model interpretation, SHAP, image analysis, near-infrared spectroscopy

## Abstract

**Introduction:**

Image and near-infrared (NIR) spectroscopic data are widely used for constructing analytical models in precision agriculture. While model interpretation can provide valuable insights for quality control and improvement, the inherent ambiguity of individual image pixels or spectral data points often hinders practical interpretability when using raw data directly. Furthermore, the presence of imbalanced datasets can lead to model overfitting and consequently, poor robustness. Therefore, developing alternative approaches for constructing interpretable and robust models using these data types is crucial.

**Methods:**

This study proposes using preprocessed data—specifically, morphological features extracted from images and chemical component concentrations predicted from NIR spectra—to build multiclass identification models. Combined kernel SVM based models were proposed to identify the rice variety and cultivation region of tobacco. The determination of kernel parameters and percentage of different types of kernel functions were accomplished by PSO, which make the approach self-adaptive. Feature importance and contribution analyses were conducted using Shapley additive explanations (SHAP).

**Results:**

The resulting models demonstrated high robustness and accuracy, achieving classification success rates of 97.9 and 97.4% via n-fold cross validation on rice and tobacco datasets, respectively, and 97.7% on an independent test set (tobacco dataset 2). This analysis identified key variables and elucidated their specific contributions to the model predictions.

**Discussion:**

This study expands the applicability of image and NIR spectroscopic data, offering researchers an effective methodology for investigating factors crucial to the quality control and improvement of agricultural products.

## 1 Introduction

Objective data analysis and machine-learning techniques have been widely employed to construct pattern recognition and regression models in agriculture. Applications include yield prediction (Fita et al., 2025), chemical composition analysis (Rawal et al., 2024), disease and pest diagnosis (Joshi et al., 2024), and soil and land management (Naeimi et al., 2024). Additionally, various chemometric methods have been investigated and applied to achieve high accuracy in agricultural analyses (Stefanov et al., 2010; Jamwal et al., 2021; Xu et al., 2023). These research efforts have significantly enhanced the accuracy and efficiency of relevant tasks while reducing associated costs.

Among the various data-acquisition methods, image analysis and near-infrared (NIR) spectroscopy are commonly used owing to their non-destructive and efficient nature (Antolínez García and Cáceres Campana, 2023; Tian et al., 2023; Zhang et al., 2024). However, most previous studies focusing on these data types have not included model interpretation. A significant challenge is that the direct meaning of individual image pixels, raw spectral data points, or features derived from dimensionality reduction techniques can be ambiguous, thereby hindering practical model interpretation. For the data pre-treatment, instead of giving data straightforward meaning, many research forced on the images recombination and dimensionality reduction for the purpose of increasing the accuracy and robustness of model. Previous studies have demonstrated that morphological features extracted from images can be used to establish identification models for various subjects, including rice and dolphins (Cinar and Koklu, 2019; Sheng et al., 2023). In the field of modeling for medical purpose, Chen et al. (2025) proposed a feature reconstruction method to reconstruct raw features from Conical Beam CT images to eventually detect cleft lip and palate. Furthermore, NIR spectra provide rich structural information of samples, resulting from multiplicative and ensemble absorption of X-H vibrations within hydrogen-containing functional groups (Xiao et al., 2023). Multiple chemical components in crops or fruits can be quantitatively predicted from NIR spectra (Zushi et al., 2025; Wang et al., 2023; Rawal et al., 2024). The straightforward meaning was signed to the data by feature extraction technique and chemical composition prediction, which could provide fundamental of conducting model interpretation method on those processed data.

Model interpretation provides valuable information crucial for controlling or improving the quality of agricultural products. The development of interpretable machine learning has led to the emergence of several methods, including permutation feature importance (Fisher et al., 2019), local interpretable model-agnostic explanations (Ribeiro et al., 2016), and Shapley additive explanations (SHAP) (Lundberg and Lee, 2017). Among these, SHAP has a solid theoretical foundation based on cooperative game theory, offering unique advantages such as fairness guarantees and the ability to provide contrastive explanations (Molnar, 2024). Consequently, it has garnered significant research attention and has been applied in diverse fields, including revealing causes of citrus fruit cracking (Abekasis et al., 2024), visually explaining liver microsomal stability models (Long et al., 2024), and facilitating feature selection in rolling-bearing fault diagnosis (Santos et al., 2024). It has also been used in feature selection in modeling for diagnosis and clinical decisions (Kha et al., 2021).

In machine learning, deep learning approaches, particularly convolutional neural networks (CNNs), have undergone rapid development and achieved impressive performance on various tasks (Luo et al., 2024). However, it is generally accepted that effectively training CNNs requires a substantial amount of data (Xu et al., 2019; Thanapol et al., 2020). However, in many practical research scenarios, available datasets are limited in size and often imbalanced. Compared with CNNs, support vector machines (SVMs) offer distinct advantages in handling smaller datasets (Han et al., 2021; Guan et al., 2021). SVM, originally proposed by Vapnik (1995), is based on the principles of Vapnik**–**Chervonenkis (VC) dimension theory and Structural Risk Minimization. It achieves good generalizability by striking an optimal balance between model complexity and learning capability, even with limited samples (Li et al., 2024). SVM is also suitable for high-dimensional feature space. Khanh Le et al. (2023) created a SVM based pipeline, including χ2 and recursive feature elimination as feature selection method, LDA as dimensionality reduction method, to predict protein crystallization propensity. Another key factor contributing to the popularity of SVM is its ability to model complex non-linear relationships through the use of appropriate kernel functions (Zhang and Wang, 2011). Consequently, the selection, construction, and optimization of suitable kernel functions and associated strategies have become active research areas (Song et al., 2008; Taqvi et al., 2022). Kernel functions can be broadly categorized into global kernels, known for their strong generalizability (e.g., linear (LKF), polynomial (PKF), and sigmoid (SKF) kernels), and local kernels, recognized for strong learning ability (e.g., radial basis function (RBF) kernel). Different kernel functions possess unique characteristics suitable for different data structures (Wang and Fang, 2019). However, because the underlying features of a dataset are often unknown beforehand, selecting the most suitable kernel function can be challenging. One approach to constructing potentially superior kernel functions involves using optimization algorithms to linearly combine multiple kernel types. This strategy aims to leverage the respective advantages of different kernels, potentially leading to enhanced model performance. It has been successfully employed for hyperspectral imagery classification (Lin & Yan, 2015), asset price prediction (Zhu et al., 2022), face recognition (Hu et al., 2022), and wind speed prediction (Tian, 2020).

This study aims to construct multiclass identification models and provide practical model interpretation using preprocessed data derived from commonly used agricultural sensing techniques. To achieve this, a morphological feature dataset for rice, originally extracted from images, was obtained from previous studies (Koklu et al., 2021; Cinar and Koklu, 2022). Additionally, two imbalanced tobacco datasets were collected, and their chemical compositions were predicted from corresponding NIR spectra using previously established chemometric models (Liang et al., 2022; Guo et al., 2023). This study employs a combined kernel function in SVM incorporating both linear and non-linear, as well as global and local kernels. Four kernel parameters and three contribution percentages within the combined kernel were optimized via particle swarm optimization (PSO) at the same time, which made this approach a self-adaptive kernel method. The resulting multiclass identification models demonstrated both strong learning and generalization capabilities. Crucially, compared to raw image or spectral data, these preprocessed features are inherently more interpretable. We employed SHAP analysis, summary and dependence plots to illustrate how different features influence class identification.

Based on the rapidly developing of the feature extraction technique and the NIR based researches of chemical composition prediction, we posit that our study—utilizing interpretable, preprocessed data derived from these techniques—is transferable and extends the applicability of image analysis and NIR spectroscopy. Moreover, models based on these interpretable preprocessed data hold significant potential for identifying key factors influencing the quality control and improvement of agricultural products.

## 2 Materials and methods

**Figure 1:**
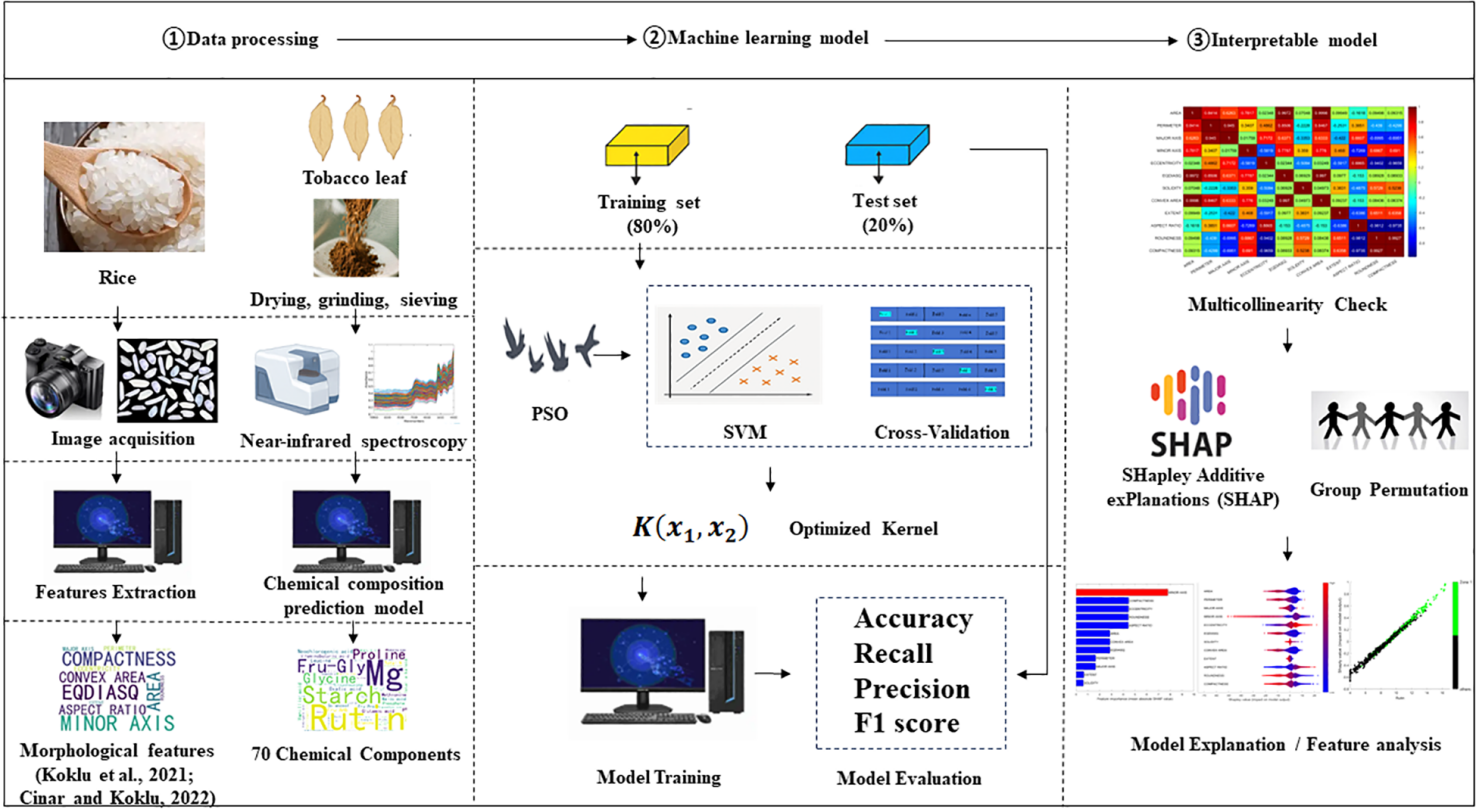
Flowchart illustrating the overall research workflow.

A study workflow is illustrated in **Figure 1**. In summary, attempts were made to combine images/NIR data, combined kernel SVM, PSO and SHAP. Firstly, data with straight forward meaning was extracted from original images/NIR data. Training set and test set was randomly separated. Then the Cross Validation (CV) was conducted on training set to determine the parameters and percentages of combined kernel. SVM model was trained with the optimized kernel. Multicollinearity between variables was checked before conducting SHAP. Group permutation was applied in SHAP to give final model interpretation. The details of each step are described in following sections.

### 2.1 Samples and data collection

The rice dataset employed in this study was obtained from previous research (Koklu et al., 2021; Cinar and Koklu, 2022) and is publicly available (https://www.muratkoklu.com/datasets/). It comprises 75,000 images of five distinct rice varieties (Arborio, Basmati, Ipsala, Jasmine, and Karacadag), with 15,000 images acquired for each variety. In this study, only the 12 morphological features, out of 106 features in original research, were used. Compared to the 90 color features, the meaning of morphological features is straightforward. The 4 shape features are a combination of certain features from the 12 morphological features.

Two distinct tobacco datasets were collected from two tobacco companies without prior sample screening based on quality attributes. In both datasets, comprising Chinese domestic tobacco samples, the class labels corresponded to eight main cultivation regions (Luo et al., 2019). Non-domestic samples were labeled according to their country of origin. The number of classes (regions or countries) exceeded ten in both datasets. Furthermore, both exhibited significant class imbalance, with disparate numbers of samples per class (details provided in **Table 1**).

**Table 1:** Number of samples per cultivation region in the tobacco datasets.

Zone	Dataset 1	Dataset 2
Domestic Zone 1	363	175
Domestic Zone 2	74	40
Domestic Zone 3	8	25
Domestic Zone 4	56	164
Domestic Zone 5	139	17
Domestic Zone 6	65	31
Domestic Zone 7	7	—
Domestic Zone 8	7	22
Brazil	48	71
Zimbabwe	30	61
America	—	20
Zambia	—	12
Total	797	638

Tobacco samples were handled following the procedures described previously (Liang et al., 2022; Guo et al., 2023). All tobacco samples were dried in a drying room at 40°C for 1−3 days, ground to a certain granularity using a whirlwind grinding mill, and sieved through a 60-mesh sieve. The moisture content of the samples ranged between 6 and 8% and was analyzed by the oven-drying method. NIR spectra were recorded for all tobacco samples using an Antaris II NIR spectrophotometer (Thermo Electron Co., USA). Measurements were performed in triplicate, and each measurement comprised 64 co-added scans recorded at a resolution of 8 cm^−1^ in the wavenumber range of 4000−10000 cm^−1^. Chemical composition data for these samples were obtained using pre-established chemometric models that predict compound concentrations from NIR spectra (Liang et al., 2022; Guo et al., 2023; Li et al., 2025). According to Liang’s report, the average R^2^ of routine chemicals, polyphenolic compounds, organic acids, amino acids, Amadori compounds, and other compounds for the EDM-PLS models were 0.949, 0.88, 0.862, 0.867, 0.945, and 0.891, respectively. The specific chemical compounds included in the analysis are listed in **Table 2**.

**Table 2:** Chemical components (n = 70) measured in the tobacco datasets.

No.	Type	Compound name	Amount
1	Routine chemicals	Total sugar, Reducing sugar, Total alkaloid, Total N, Potassium ion(K), Chloridion (Cl), Starch	7
2	Ion	Sulfate, Phosphate, Calcium(Ca), Magnesium (Mg),	4
3	Polyphenolic compounds	Neochlorogenic acid, Chlorogenic acid, Cryptochlorogenic acid, Scopoletin, Rutin,	5
4	Organic acids	Oxalic acid, Propanedioic acid, Succinic acid, Malic acid, Citric acid, Vanillic acid, Myristic acid, Palmitic acid, Oleic acid and Linolenic acid, Linoleic acid, Stearic acid, arachidic acid	12
5	Amino acid	Aspartic acid, L-Threonine, Serine, L-Asparagine, Glutamic acid, Glutamine, Glycine, Alanine, Valine, Cystine, Methionine, L-isoleucine, Leucine, Tyrosine, Phenylalanine, γ-aminobutyric acid, Lysine, Histidine, Tryptophan, Arginine, Proline	21
6	Amadori compounds	N-(1-Deoxy-d-glucose-1-yl) Ammonia (Glu-An), N-(1-deoxy-D-fructos-1-yl) aminobutyric(Fru-Amb), N-(1-deoxy-D-fructos-1-yl) Histidine(Fru-His), N-(1-deoxy-D-fructos-1-yl) Proline(Fru-Pro), N-(1-deoxy-D-fructos-1-yl) Valine(Fru-Val), N-(1-deoxy-D-fructos-1-yl) Threonine(Fru-Thr), N-(1-deoxy-D-fructos-1-yl) Glycine(Fru-Gly), N-(1-deoxy-D-fructos-1-yl) Alanine(Fru-Ala), N-(1-deoxy-D-fructos-1-yl) Asparagine(Fru-Asn), N-(1-deoxy-D-fructos-1-yl) Asparticacid(Fru-Asp), N-(1-deoxy-D-fructos-1-yl) Glutarnine(Fru-Gln), N-(1-deoxy-D-fructos-1-yl) Glutamicacid(Fru-Glu), N-(1-deoxy-D-fructos-1-yl) Isoleucine(Fru-Ile), N-(1-deoxy-D-fructos-1-yl) Leucine(Fru-Leu), N-(1-deoxy-D-fructos-1-yl) Tyrosine(Fru-Tyr), N-(1-deoxy-D-fructos-1-yl) Phenylalanine(Fru-Phe), N-(1-deoxy-D-fructos-1-yl) Tryptophan(Fru-Trp)	17
7	Others	Dichloromethane extraction, pH value, Solanesol, Neophytadiene	4
Total			70

### 2.2 Combined kernel and optimization

In the non-linearly separable data, SVM utilizes the kernel trick: the input data are mapped into a higher-dimensional feature space via a kernel function, where linear separation becomes feasible. The one-vs-all (OVA) approach was adopted, where M individual binary SVM classifiers are trained (M is the total number of classes), each separating one class from all the others. This choice was guided by reports suggesting potentially higher accuracy than the OVO strategy in certain contexts (Taqvi et al., 2022). A key aspect of this study is the use of a combined kernel function. Specifically, LKF, PKF, and RBF kernels (detailed in **Table 3**)—encompassing linear/non-linear and global/local types—are linearly combined as follows (**Equation 1**):

**Table 3:** Kernel functions used for SVM modeling in this study.

Kernel	Formula
LKF	$\text{K}\left(x_{1},x_{2}\right)=x_{1}^{T}x_{2}$
PKF	$\text{K}\left({\text{x}}_{1},{\text{x}}_{2}\right)={\left(\text{a}\cdot {\text{x}}_{1}^{T}{\text{x}}_{2}+\text{b}\right)}^{q}$
RBF	$\text{K}\left({\text{x}}_{1},{\text{x}}_{2}\right)=\text{exp}\left(\frac{-\|\left|{\text{x}}_{1}-{\text{x}}_{2}\right|{\|}^{2}}{{\text{σ}}^{2}}\right)$


(1) $$K\left(x_{1},x_{2}\right)=p_{1}*x_{1}^{T}x_{2}+p_{2}*{\left(a\cdot x_{1}^{T}x_{2}+b\right)}^{q}+p_{3}*exp\left(\frac{-\|\left|x_{1}-x_{2}\right|{\|}^{2}}{\sigma^{2}}\right)$$

The optimal kernel percentages ( $p_{1},p_{2},p_{3}$ ) and kernel-specific parameters ( $\text{a},\;b,\;q,\;\sigma^{2}$ ) were determined via PSO. PSO is a swarm intelligence algorithm developed by Eberhart and Kennedy (1995), inspired by the social foraging behavior of bird flocks. Owing to its simplicity, robustness, and efficiency, PSO has undergone considerable development and found widespread application (Zhang and Teng, 2009). In PSO, a population of particles (representing potential solutions) is initialized randomly within the search space, and each is evaluated by the fitness function at its current location. During each iteration, each particle adjusts its position based on its current velocity (inertia), its own best-known positions, and the best-known position found by the entire swarm, with some random perturbations as follow (**Equations 2** and **3**):


(2) $$v_{i}^{k+1}=w*v_{i}^{k}+c_{1}*r_{1}*\left(pbest_{i}^{k}-x_{i}^{k}\right)+c_{2}*r_{2}*\left(gbest_{i}^{k}-x_{i}^{k}\right)$$

(3) $$x_{i}^{k+1}=x_{i}^{k}+v_{i}^{k+1}$$

where $v_{i}^{k}$ and $x_{i}^{k}$ represent the velocity and position of the *i-*th particle at iteration *k*. The parameters c_1_ and c_2_ are acceleration factors, both set to 0.8 in this study. Additionally, r_1_ and r_2_ are random numbers uniformly distributed in the range [0, 1]. A time-decreasing inertia weight (*w*) strategy was employed to balance global and local search capabilities during optimization. CV is a standard technique used to assess the generalizability of classification models (Ong et al., 2005; Koklu et al., 2021). In this study, the average classification accuracy obtained from *k*-fold CV (with *k* typically set to 5 or 10) was used as the fitness function for the PSO algorithm. This directs the optimization process towards finding kernel parameters and weightings that yield models with strong generalizability. The kernel parameters and their percentages were optimized via PSO at the same time, which made this approach a self-adaptive kernel method.

### 2.3 Model evaluation

In this study, the model evaluation approach varied across different datasets. For the rice dataset, ten-fold CV was applied to ensure that the results were comparable with previous research findings. For Tobacco Dataset 1, a five-fold CV was used to evaluate the models as the sample sizes for a few cultivation regions were smaller than ten. For Tobacco Dataset 2, evaluation involved randomly selecting approximately 20% of the samples as a test set, with the remaining samples constituting the training set. A five-fold CV was conducted on this training set to determine the optimal kernel parameters. Subsequently, the model was trained using the determined parameters, and the predictive accuracy on the test set was used to evaluate its performance.

### 2.4 Shapley value and SHAP

The Shapley value, coined by Shapley (1997), assigns payouts to features depending on their contribution to the model’s prediction (total payout). It represents the average marginal contribution of a feature’s value across all possible feature combinations. The Shapley value was estimated using the approximation method detailed in **Algorithm 1**, employing Monte-Carlo sampling as proposed by Štrumbelj and Kononenko (2014). SHAP (Lundberg and Lee, 2017), by using Shapley values, provides global interpretation methods derived from aggregations of these individual Shapley values.

Algorithm 1 **Approximating the contribution of the *j-*th feature for model *f*.**


For 
m=1,…,M
:
• Randomly select an instance 
z
 from the data matrix 
X

• Generate a random permutation o of the feature indices
• Order instance 
$\text{x}:\;x_{0}=\left(x_{\left(1\right)},\ldots ,x_{\left(j\right)},\ldots ,x_{\left(p\right)}\right)$
.
• Order instance 
$\text{z}:\;z_{0}=\left(z_{\left(1\right)},\ldots ,z_{\left(j\right)},\ldots ,z_{\left(p\right)}\right)$
.
• Construct two new instances.
• With 
$\text{ j}:\;x_{+j}=\left(x_{\left(1\right)},\ldots ,x_{\left(j-1\right)},x_{\left(j\right)},z_{\left(j+1\right)},\ldots ,z_{\left(p\right)}\right)$
.
• Without 
$\text{j}:\;x_{-j}=\left(x_{\left(1\right)},\ldots ,x_{\left(j-1\right)},z_{\left(j\right)},z_{\left(j+1\right)},\ldots ,z_{\left(p\right)}\right)$
.
• Compute marginal contribution: 
$\phi_{j}^{m}=\hat{f}\left(x_{+j}\right)-\hat{f}\left(x_{-j}\right)$

End for
• Compute the average Shapley value: 
$\phi_{j}\left(\text{x}\right)=\frac{1}{M}\sum_{m=1}^{M}\phi_{j}^{m}$

Here, x is the selected instance being explained, j is the index of the feature whose contribution is being estimated, and M is the number of iterations, which was set to 300 in this study. Meanwhile, group permutation was conducted for the variables if they were highly correlated (|R|>0.8) with j.

## 3 Results and discussion

### 3.1 Rice variety identification: model construction and evaluation

Rice is one of the most widely produced and consumed cereal crops globally. The quality attributes of rice, such as cooking properties, aroma, and taste, are closely related to its variety.

First, a linear SVM was used to construct an identification model, achieving an average total accuracy of 97.3% in ten-fold CV (**Supporting Information Table S1**). Subsequently, the PKF was evaluated, with the “PolynomialOrder” parameter set to 2 within the “fitcsvm” function. This approach yielded an improved average total accuracy of 97.9% (**Table 4**), and the accuracy for each variety also improved. Considering that only 12 morphological features were used in this study, as opposed to the 106 features employed in the original research, this performance level is considered acceptable.

**Table 4:** Classification accuracy results (%) of ten-fold CV on the rice dataset.

Variety	Fold1	Fold2	Fold3	Fold4	Fold5	Fold6	Fold7	Fold8	Fold9	Fold10	Average
Basmati	97.1	97.4	96.9	98.2	97.8	98.1	97.5	97.8	97.2	97.6	97.5
Arborio	97.1	96.9	96.9	96.5	96.0	96.8	96.9	97.2	96.5	96.6	96.7
Jasmine	98.0	98.3	98.6	98.3	97.7	98.0	98.4	97.9	98.1	98.5	98.2
Ipsala	99.4	99.6	99.2	99.7	99.3	99.4	99.5	99.7	99.3	99.6	99.5
Karacadag	98.4	97.4	97.4	96.7	98.5	98.1	97.4	98.1	97.7	97.8	97.8
Total	98.0	97.9	97.8	97.9	97.9	98.1	97.9	98.1	97.8	98.0	97.9

### 3.2 Rice variety identification: model interpretation

Cinar and Koklu (2021) previously conducted variable analysis on this dataset using analysis of variance, chi-squared test, and gain ratio, providing an importance order for effective features. Although such statistical information is valuable, it does not directly interpret the classification model itself. This study applied the SHAP approach, using one OVA model (Basmati vs. others) as an illustrative example.

**Figure 2:**
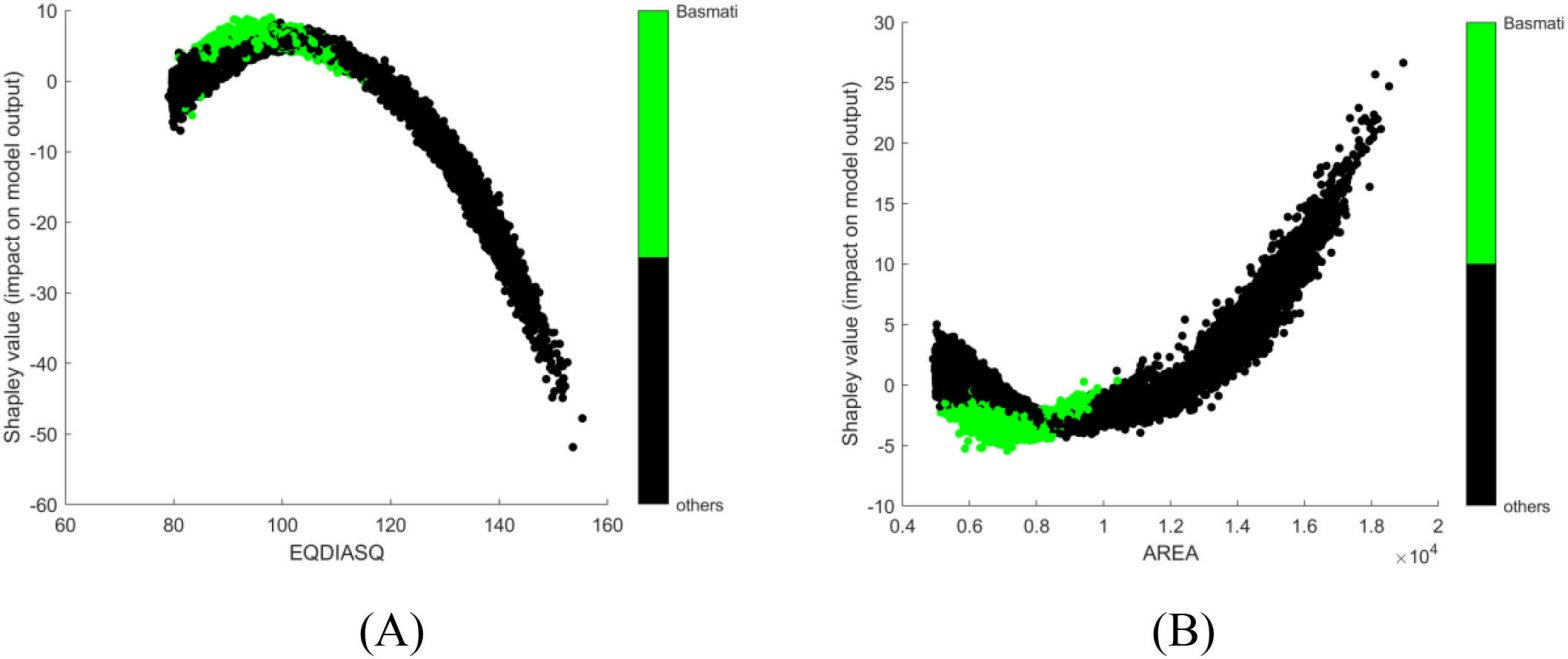
SHAP dependence plots, no corrective measures are implemented for multicollinearity: **(A)** EQDIASQ; **(B)** AREA.

**Figure 3:**
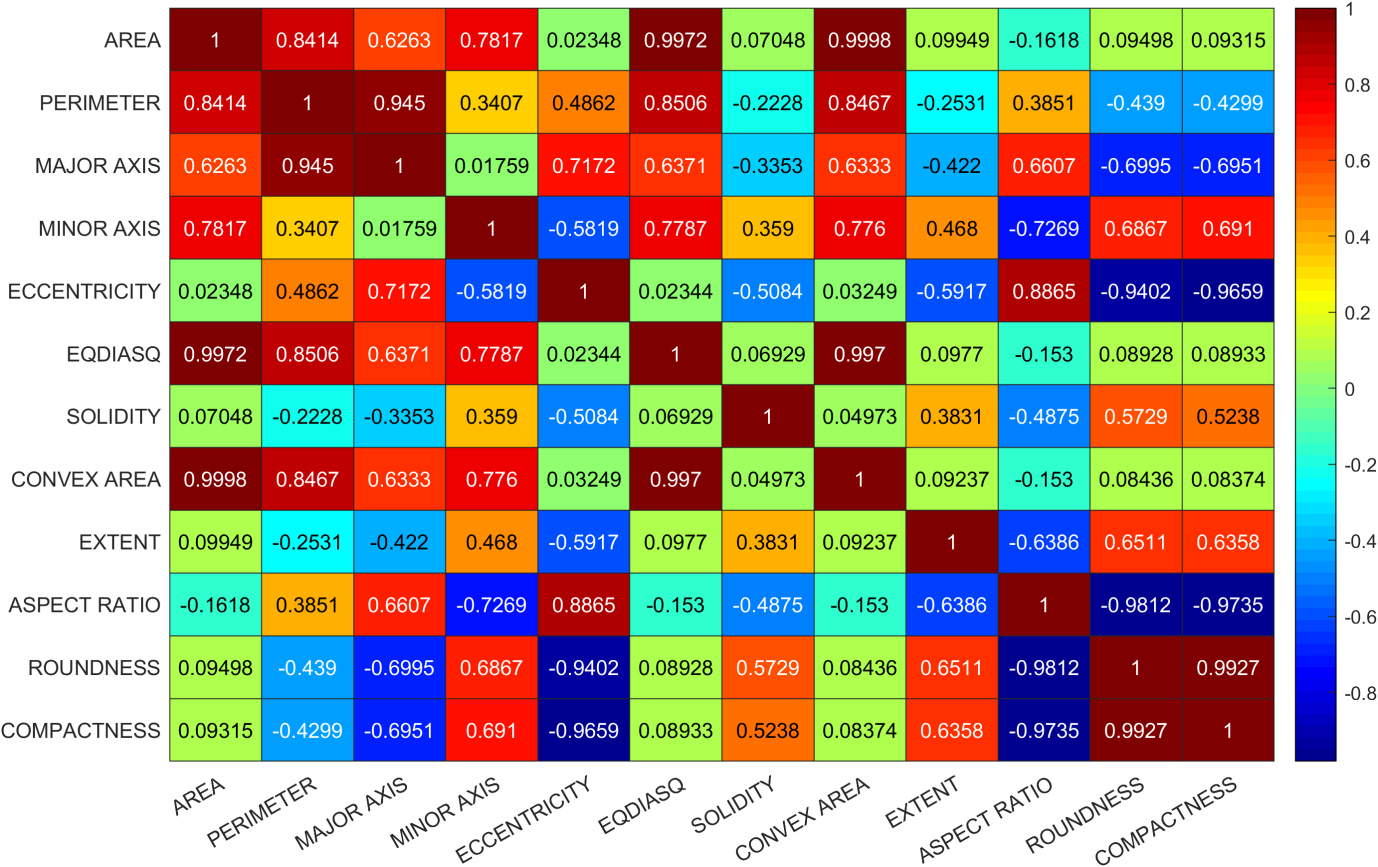
Heatmap of correlation with correlation coefficient between variables.

In the first trial, no corrective measures are implemented in SHAP to deal with multicollinearity issue and obvious conflict was observed in interpretation. For instance, EQDIASQ exhibited a quadratic relationship between the variable value and the corresponding Shapley value (**Figure 2A**). The mean EQDIASQ values of Ipsala, Jasmine, and Karacadag were either higher or lower than those of Basmati and Arborio (**Table 5**). It is easy to accept the interpretation that the model would tent to output lower Shapley values (indicating less support for Basmati) if the EQDIASQ value is excessively high or low relative to the typical Basmati range. However, Area, which is highly correlated to EQDIASQ (**Figure 3**, heatmap of correlation with correlation coefficient between variables), showed an opposite quadratic relationship (**Figure 2B**). A similar pattern was observed in the dependence plot for CONVEX AREA. This counterintuitive interpretation could be caused by high correlations between those variables.

**Table 5:** Mean values of morphological variables for the five rice varieties.

Variable	Arborio	Basmati	Ipsala	Jasmine	Karacadag
AREA	7531.717	7563.938	14048.645	6267.308	6484.379
PERIMETER	339.852	426.906	476.498	347.781	299.810
MAJOR AXIS	137.585	202.336	197.071	157.076	114.959
MINOR AXIS	70.459	48.494	91.817	50.951	72.426
ECCENTRICITY	0.857	0.970	0.884	0.945	0.774
EQDIASQ	97.790	97.975	133.549	88.545	90.797
SOLIDITY	0.977	0.970	0.977	0.972	0.983
CONVEX AREA	7712.890	7797.524	14373.349	6442.758	6597.790
EXTENT	0.683	0.504	0.663	0.590	0.726
ASPECT RATIO	1.958	4.194	2.153	3.089	1.591
ROUNDNESS	0.818	0.522	0.776	0.642	0.906
COMPACTNESS	0.711	0.485	0.678	0.565	0.791

No units were provided in the original data extracted from images.

**Figure 4:**
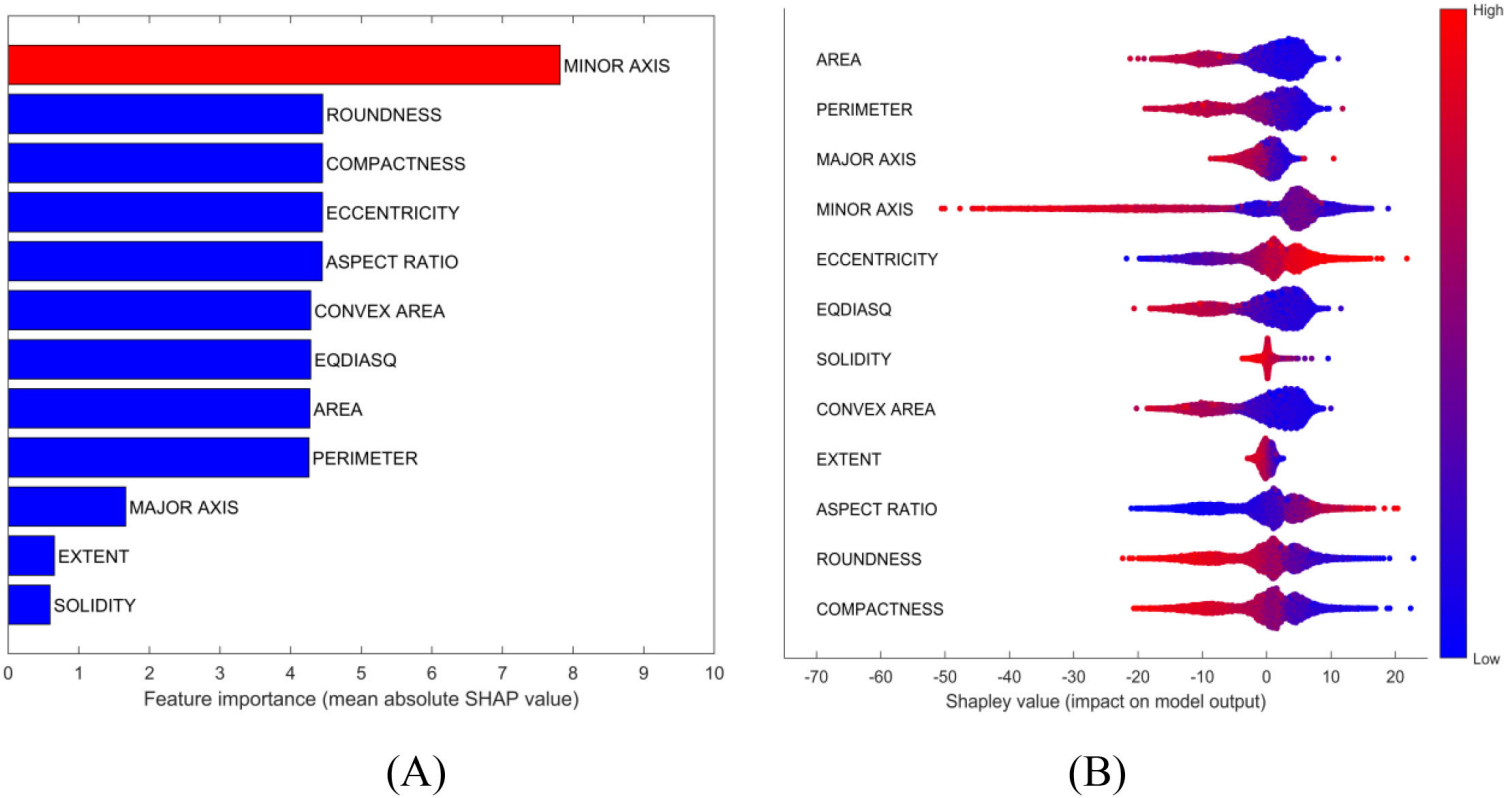
Model interpretation plots for the rice dataset: **(A)** Feature importance ranking based on mean absolute SHAP values; **(B)** SHAP summary plot illustrating feature impacts, where red and blue points correspond to high and low feature values, respectively.

Potential solutions to address interpretation issues arising from multicollinearity include permuting correlated features together to obtain a mutual Shapley value or employing conditional sampling (Molnar, 2024). Therefore, group permutation was implemented in this study, which means that the highly correlated variables will be permuted together. SHAP uses mean absolute Shapley values to evaluate variable importance (**Figure 4A**). For each variables, its absolute Shepley value from all the samples will averaged to give the length of bar in figure. The SHAP summary plot gives global view of contributions of the variables (**Figure 4B**). In the summary plot, each variable is represented by a dotted line along the horizontal axis. Red dots indicate high values of the variable in a given sample, whereas blue dots represent low values. A higher Shapley value signifies that the variable makes a positive contribution toward classifying the sample into the target class (Basmati in this example), whereas a lower value indicates a contribution towards classifying it as one of the other varieties. From the perspective of the constructed model, MINOR AXIS was identified as the most important variables for identifying Basmati rice, and it showed a negative contribution towards the Basmati classification. The basically same importance and contribution were signed to the correlated variables. ROUNDNESS, COMPACTNESS, ECCENTRICITY and ASPECT RATIO were signed as the second important variables as a group.

**Figure 5:**
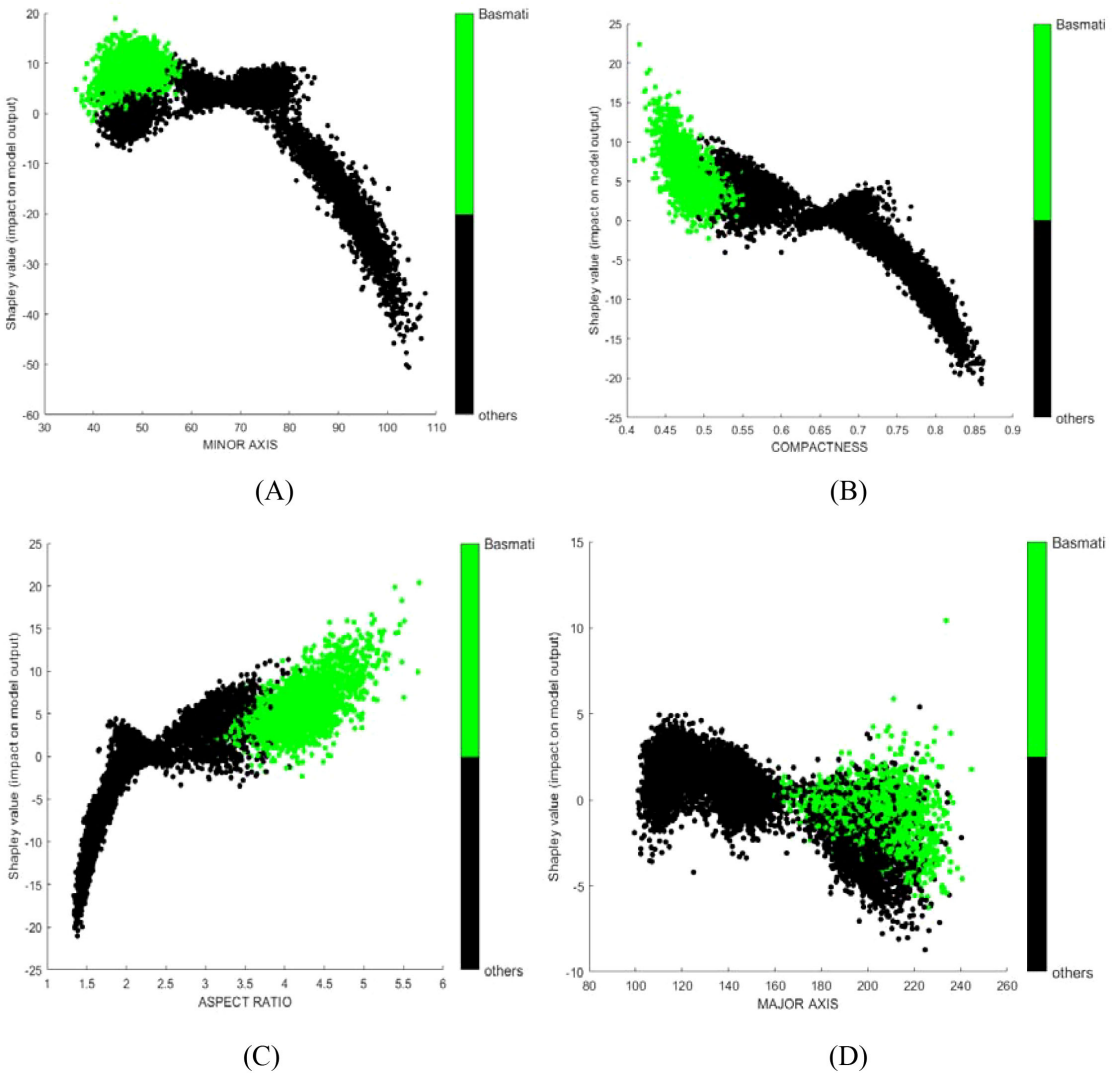
SHAP dependence plots for selected features from the rice dataset model: **(A)** MINOR AXIS; **(B)** ROUNDNESS; **(C)** ASPECT RATIO; **(D)** MAJOR AXIS. Green points represent samples of the target class; black points represent samples from other classes.

SHAP dependence plots were used to further investigate how individual variables affect the identification outcome. For instance, MINOR AXIS consistently made a negative contribution in the model (**Figure 5A**), which aligns with the observation that the mean value of MINOR AXIS for Basmati is the lowest among all rice varieties considered (**Table 5**, mean value of each variables of each class). COMPACTNESS and ASPECT RATIO showed negative and positive contribution (**Figures 5B, C**), respectively, which also aligns with their mean value. Some variables, like MAJOR AXIS showed very limited contribution (**Figure 5D**).

### 3.3 Cultivation region identification: model construction and evaluation

The quality of many agricultural products, including fruits, mushrooms, tobacco, and traditional Chinese medicines, is significantly affected by their cultivation regions (Li et al., 2018; Wei et al., 2018; Kim et al., 2020; Jiang et al., 2020; Li et al., 2022).

Given that the tobacco datasets were limited in size, imbalanced, and included multiple region classes, linear methods were evaluated first. Stepwise Fisher linear discriminant analysis (LDA) resulted in serious overfitting for Domestic Zones 3, 7, and 8. By contrast, linear SVM showed higher or similar CV accuracy, implying better generalizability in this context (**Supporting Information Table S2**, complete CVs in **Supplementary Tables S3, S4**). LDA relies heavily on variance and scatter matrices (Ghojogh et al., 2022), and small sample sizes may not provide stable estimations for these parameters.

Because potential non-linear relationships between the chemical variables and region classes might exist, kernel SVM models were subsequently established. The kernel function parameters for each kernel type were set manually through multiple trials, and the best results are listed in **Supplementary Tables S5–S7**. The PKF generally demonstrated better generalizability than the other kernel functions tested. Notably, satisfactory average accuracies were achieved even for groups with small sample sizes. Following these initial explorations, the combined kernel approach was implemented. A performance comparison of combined kernel SVM against models using single, manually tuned kernel functions is presented in **Table 6**. The kernel parameters optimized by PSO are listed in **Table 7**. Combined kernel SVM achieved the highest, or equally highest, average CV accuracies for all individual regions and the highest overall accuracy. The confusion matrix, including F1-score, precision, recall, and five-fold CV results for combined kernel SVM are provided in Supporting Information (**Supplementary Tables S8, S9**). The over-sampling was also conducted to fix the imbalance issue. However, limited improvement was achieved in LDA and even lower accuracy was achieved in SVM (details and results in **Supporting Information, Table S11**). The paired samples t-test of different methods can be found in Supporting Information (**Supplementary Table S12**).When comparing with other methods, the combined kernel SVM without over-sampling showed significant difference in most case.

**Table 6:** Average classification accuracies (%) of five-fold CV on tobacco dataset 1.

Zone label	LKF	PKF	RBF	SKF	Combined kernel SVM
Domestic Zone 1	98.1	99.2	99.5	99.2	99.7
Domestic Zone 2	84.0	86.7	73.1	25.8	88.0
Domestic Zone 3	40.0	80.0	50.0	0.0	80.0
Domestic Zone 4	94.8	98.3	96.7	96.5	98.3
Domestic Zone 5	98.6	98.6	97.9	95.0	98.6
Domestic Zone 6	95.4	93.8	93.8	90.8	95.4
Domestic Zone 7	100.0	100.0	100.0	80.0	100.0
Domestic Zone 8	90.0	90.0	70.0	70.0	90.0
Brazil	94.0	96.0	92.0	96.0	96.0
Zimbabwe	96.7	100.0	100.0	100.0	100.0
Total Accuracy	95.4	96.9	94.9	89.2	97.4
*Note*: Kernel parameter	—	a = 1/65, b = 1.5, q = 3	${\text{σ}}^{2}$ = 32	a = 1/65, b = -1.2	In **Table 7**

**Table 7:** Optimized parameters for the combined kernel function obtained via PSO.

Kernel percentage (%)			Kernel parameters			
			PKF			RBF
LKF	PKF	RBF	a	b	q	$\sigma^{2}$
0.0	44.5	55.5	65	1.5	3	32

To further evaluate the combined kernel SVM method, an independent test set was randomly selected from Tobacco Dataset 2. The combined kernel SVM approach, including parameter optimization via CV on the training set, was applied to the training set. The resulting model was then used to predict the classes of the test set samples. The results are presented in Supporting Information (**Supplementary Tables S13–S15**). The model demonstrated a high accuracy of 97.7% on the independent test set and F1-score range for each class is from 0.923 to 1. To further evaluate the robustness of model, 10 times repeating experiments was conducted to give Standard Deviation (SD) and Confidence Intervals (CI). For each trial, training set and independent test set was randomly separated. The approach mentioned above was re-conducted. The SD and CI of recall and total accuracy were listed in the **Table 8**. Generally, the model showed good accuracy and rubustness. 5 zones showed standard deviations more the 10%. 3 zones showed confidence intervals more than ±10%. The possible reason is the limited amount of samples. Therefore, it is necessary to keep collecting samples in future work. An extra dataset was collected as external test data. The Total accuracy is 95.2% and the confusion matrix, including F1-score, precision, recall, was presented in Supporting Information (**Supplementary Table S16**).

**Table 8:** Standard deviation and confidence intervals of recall and total accuracy.

Cultivation region	Repeat 1	Repeat 2	Repeat 3	Repeat 4	Repeat 5	Repeat 6	Repeat 7	Repeat 8	Repeat 9	Repeat 10	Average	Standard deviation	Confidence intervals (95%)
Domestic Zone 1	100.0%	100.0%	97.4%	100.0%	100.0%	94.9%	94.6%	100.0%	100.0%	97.6%	98.5%	2.2%	98.5% ± 1.4%
Domestic Zone 2	87.5%	85.7%	100.0%	90.0%	44.4%	83.3%	100.0%	66.7%	60.0%	100.0%	81.8%	18.9%	91.8% ± 11.7%
Domestic Zone 3	80.0%	100.0%	100.0%	100.0%	66.7%	100.0%	100.0%	80.0%	100.0%	75.0%	90.2%	13.2%	90.2% ± 8.2%
Domestic Zone 4	97.0%	100.0%	97.1%	96.4%	97.1%	100.0%	94.9%	100.0%	97.1%	100.0%	98.0%	1.9%	98.0% ± 1.2%
Domestic Zone 5	100.0%	100.0%	100.0%	80.0%	100.0%	71.4%	100.0%	100.0%	50.0%	100.0%	90.1%	17.5%	90.1% ± 10.8%
Domestic Zone 6	100.0%	100.0%	100.0%	100.0%	100.0%	100.0%	100.0%	100.0%	100.0%	100.0%	100.0%	0.0%	100.0% ± 0%
Domestic Zone 8	100.0%	80.0%	100.0%	100.0%	100.0%	66.7%	100.0%	100.0%	100.0%	83.3%	93.0%	12.0%	93.0% ± 7.4%
Brazil	100.0%	94.1%	100.0%	100.0%	100.0%	100.0%	100.0%	100.0%	100.0%	100.0%	99.4%	1.9%	99.4% ± 1.2%
Zimbabwe	100.0%	100.0%	100.0%	100.0%	100.0%	90.0%	92.9%	100.0%	100.0%	100.0%	98.3%	3.7%	98.3% ± 2.3%
America	100.0%	100.0%	100.0%	100.0%	100.0%	100.0%	100.0%	100.0%	100.0%	100.0%	100.0%	0.0%	100.0% ± 0%
Zambia	100.0%	66.7%	NaN	50.0%	75.0%	66.7%	50.0%	100.0%	100.0%	100.0%	78.7%	21.7%	78.7% ± 14.2%
Total Accuracy	97.7%	96.9%	98.4%	96.3%	93.7%	93.7%	95.4%	96.3%	96.6%	97.8%	96.3%	1.6%	96.3% ± 1.0%

NaN means no sample was selected as test set of this zone due to the random sampling.

### 3.4 Cultivation region identification: model interpretation

**Figure 6:**
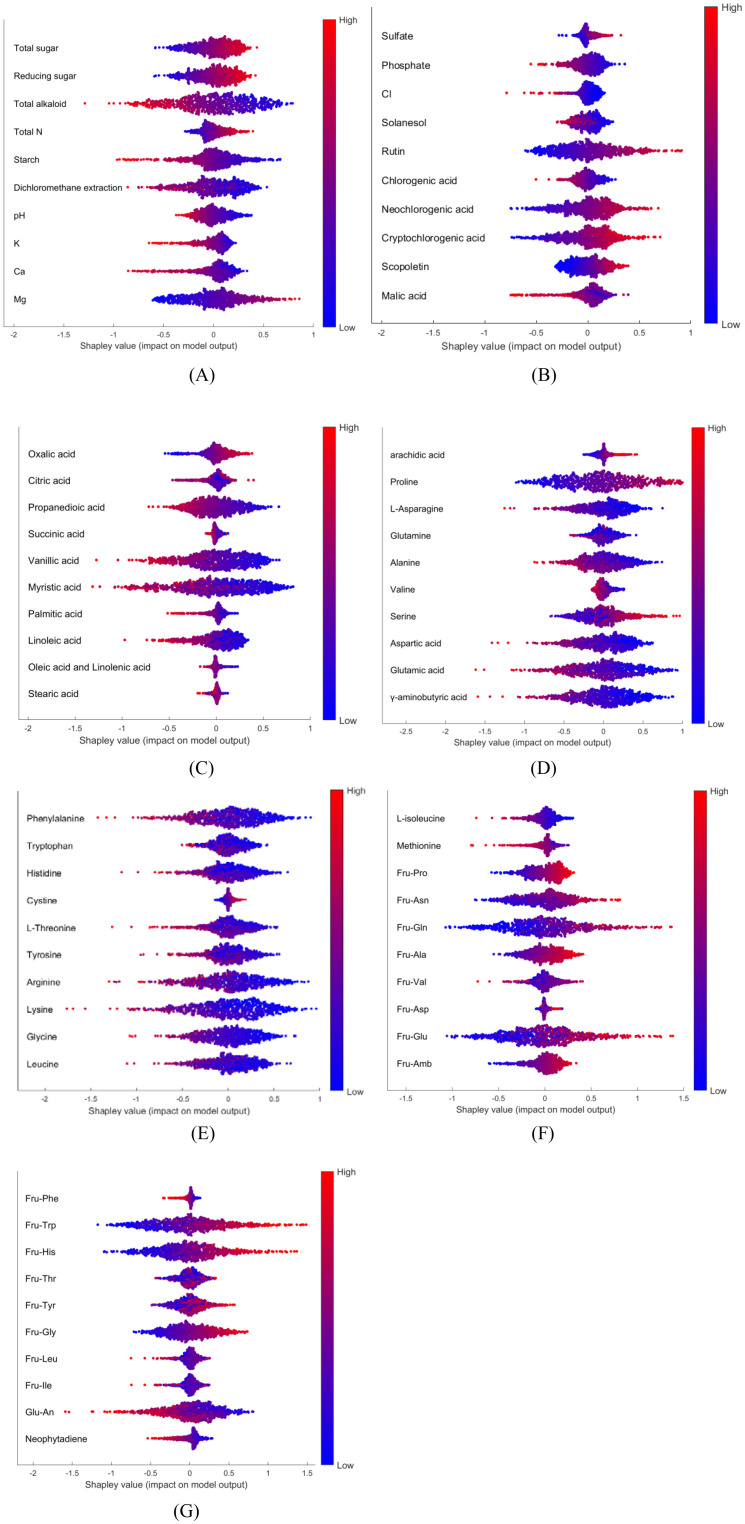
SHAP summary plot for all 70 compounds, Red and blue points correspond to high and low chemical levels, respectively.: **(A)** part1; **(B)** part2; **(C)** part3; **(D)** part4; **(E)** part5; **(F)** part6; **(G)** part7.

One OVA model (Domestic Zone 1 vs. others), constructed using Tobacco Dataset 1, was analyzed using the SHAP approach. Group permutation was also applied in this case (Full Heatmap of 70 compounds in Supporting Information, **Supplementary Figure S1**). **Figures 6A–G** presents the global SHAP analysis for selected compounds relevant to identifying samples from Domestic Zone 1 versus other regions. As depicted in **Figure 6**, certain compounds exhibit a positive contribution towards identifying Domestic Zone 1 samples, such as magnesium (Mg) and Oxalic acid. Conversely, some compounds show a negative contribution, such as starch and vanillic acid. The overlap of red and blue dots for some variables, such as succinic acid and stearic acid (**Figure 6C**), indicates that these features did not consistently contribute either positively or negatively to the model’s output across all samples.

**Figure 7:**
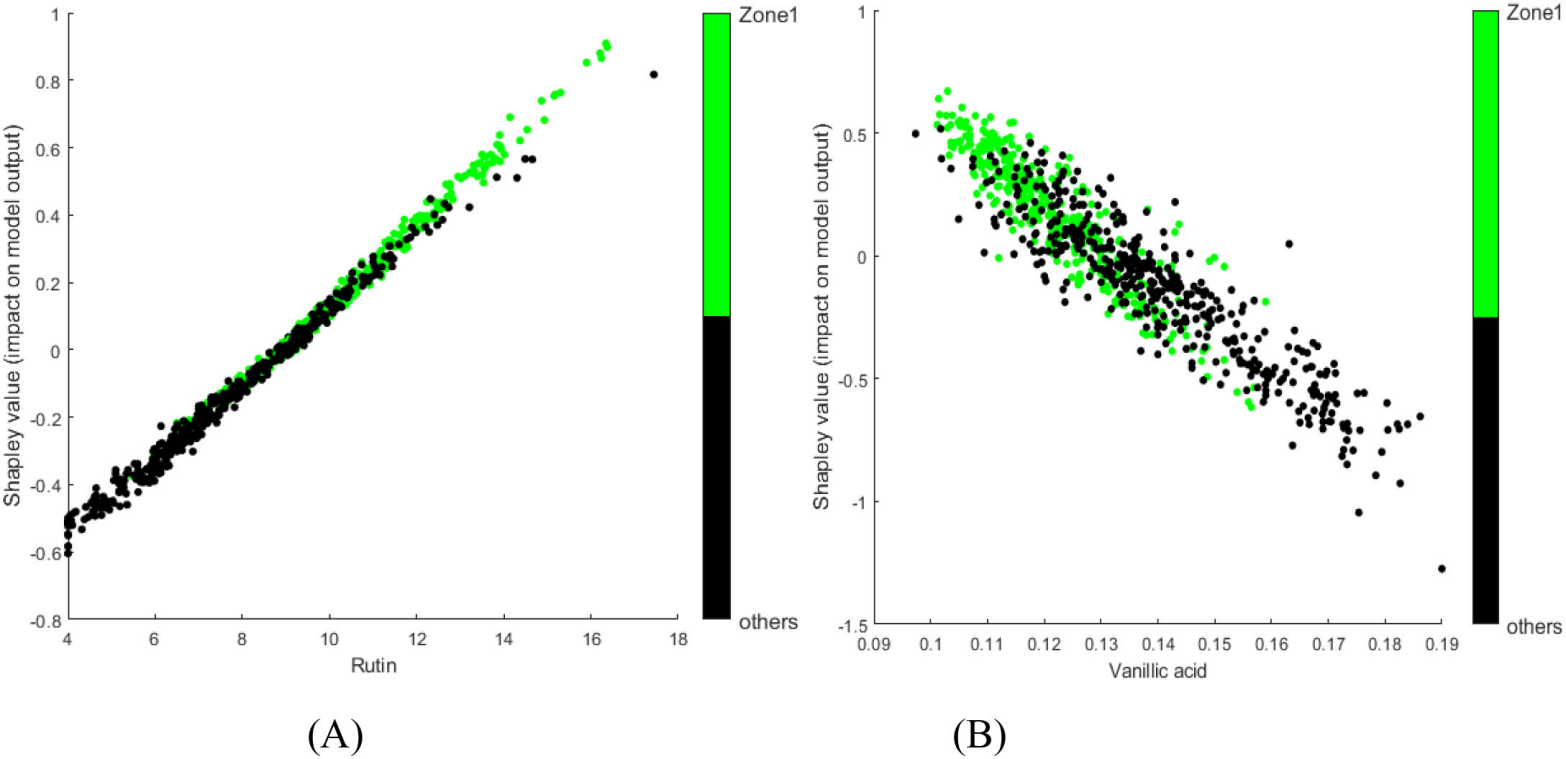
SHAP dependence plots for selected chemical components from the tobacco dataset model: **(A)** rutin; **(B)** vanillic acid. Green points represent samples from the target class; black points represent samples from other classes.

To further interpret the model, SHAP dependence plots were generated to illustrate the influence of chemical levels on the identification. **Figures 7A, B** show the SHAP dependence plots for rutin and vanillic acid, respectively, overlaid with sample classification information. Green dots represent samples from Domestic Zone 1, whereas black dots represent those from other regions. For a single variable, overlap between samples from different classes can often be observed in the middle range of chemical values, regardless of whether the overall contribution is positive or negative. However, clear trends can be observed. With increasing levels of rutin or decreasing levels of vanillic acid, a greater proportion of samples were identified as belonging to Domestic Zone 1 rather than other regions. Some previous reports provide corroborating evidence from a different perspective. The content levels of rutin, total sugar, and total N are fairly high, while the K levels are relatively low, in samples from Domestic Zone 1 (Luo et al., 2019). These chemical components showed correspondingly positive (rutin, total sugar, total N) or negative (K) contributions in our SHAP analysis (**Figures 6A, B**). Similarly, Wang et al. (2022) found that the chemical components Fru-Pro, Fru-Gln, and Fru-His are typically high in samples from Domestic Zone 1, and these components also showed clear positive contributions in our analysis (**Figures 6F, G**). Compared to traditional methods of analyzing variable differences between groups, this model interpretation approach is arguably more efficient, yielding detailed and straightforward insights into feature contributions.

Remarkably, many variables displayed a strong linear relationship between their value and their corresponding Shapley value (contribution). The dependence plots of variables exhibiting a coefficient of determination (R^2^) lower than 0.8 between the variable and Shapley values were manually verified. None of these showed clear relationship of other kind such as quadratic relationship. This observation suggests that the absolute value of the slope formed by the points in a SHAP dependence plot could serve as an alternative measure of variable importance, particularly when a clear linear trend exists. Based on this assumption, we identified the important compounds for distinguishing between samples from Domestic Zone 1 and other samples using this slope-based metric (**Table 9**). The compounds with R^2^ higher than 0.8 was listed in row 1 to 29, ordered by absolute slope value from high to low. The compounds with R^2^ lower than 0.8 was listed in row 30 to 70 and their positive/negative contribution was labeled as N/A. Considering the potentially imbalanced distribution of Shapley values across a variable’s range, this slope-based method might offer a fairer assessment of importance than the mean absolute Shapley value for certain cases.

**Table 9:** Chemical components identified as most important for distinguishing domestic zone 1 samples based on SHAP analysis.

No.	Compound	Slope	R^2^	Absolute slope value	Positive (+) or negative (-) contribution
1	Proline	2.452	0.885	2.452	+
2	γ-aminobutyric acid	-1.939	0.825	1.939	–
3	Mg	1.779	0.980	1.779	+
4	Fru-Trp	1.765	0.861	1.765	+
5	Glycine	-1.633	0.828	1.633	–
6	Fru-His	1.572	0.826	1.572	+
7	Total alkaloid	-1.566	0.911	1.566	–
8	Rutin	1.529	0.992	1.529	+
9	Vanillic acid	-1.529	0.864	1.529	–
10	Starch	-1.396	0.963	1.396	–
11	Fru-Gly	1.364	0.973	1.364	+
12	Neochlorogenic acid	1.343	0.933	1.343	+
13	Propanedioic acid	-1.277	0.898	1.277	–
14	Cryptochlorogenic acid	1.108	0.891	1.108	+
15	Total sugar	0.939	0.965	0.939	+
16	Reducing sugar	0.928	0.978	0.928	+
17	Fru-Amb	0.911	0.973	0.911	+
18	Fru-Ala	0.897	0.881	0.897	+
19	Oxalic acid	0.824	0.936	0.824	+
20	Phosphate	-0.808	0.978	0.808	–
21	pH	-0.739	0.968	0.739	–
22	L-isoleucine	-0.736	0.896	0.736	–
23	Fru-Pro	0.706	0.958	0.706	+
24	K	-0.694	0.912	0.694	–
25	Scopoletin	0.640	0.942	0.640	+
26	Chlorogenic acid	-0.595	0.934	0.595	–
27	Cl	-0.563	0.905	0.563	–
28	Total N	0.562	0.885	0.562	+
29	Palmitic acid	-0.547	0.878	0.547	–
30	Ca	-1.076	0.791	1.076	N/A
31	Neophytadiene	-0.566	0.771	0.566	N/A
32	arachidic acid	0.451	0.753	0.451	N/A
33	Linoleic acid	-0.828	0.748	0.828	N/A
34	Myristic acid	-1.655	0.736	1.655	N/A
35	L-Asparagine	-1.526	0.724	1.526	N/A
36	Cystine	0.194	0.723	0.194	N/A
37	Fru-Gln	1.595	0.722	1.595	N/A
38	Sulfate	0.282	0.706	0.282	N/A
39	Alanine	-1.215	0.682	1.215	N/A
40	Aspartic acid	-1.397	0.673	1.397	N/A
41	Glutamic acid	-1.680	0.671	1.680	N/A
42	Methionine	-0.748	0.669	0.748	N/A
43	Glu-An	-1.312	0.666	1.312	N/A
44	Fru-Glu	1.388	0.659	1.388	N/A
45	Fru-Asn	1.116	0.653	1.116	N/A
46	Lysine	-1.812	0.652	1.812	N/A
47	Phenylalanine	-1.424	0.644	1.424	N/A
48	Fru-Phe	-0.216	0.619	0.216	N/A
49	Arginine	-1.362	0.599	1.362	N/A
50	Dichloromethane extraction	-1.017	0.590	1.017	N/A
51	Malic acid	-0.682	0.576	0.682	N/A
52	Solanesol	-0.366	0.562	0.366	N/A
53	Valine	-0.350	0.539	0.350	N/A
54	Serine	0.870	0.530	0.870	N/A
55	Succinic acid	-0.106	0.443	0.106	N/A
56	Leucine	-1.051	0.440	1.051	N/A
57	L-Threonine	-0.787	0.427	0.787	N/A
58	Histidine	-0.806	0.423	0.806	N/A
59	Tyrosine	-0.666	0.351	0.666	N/A
60	Fru-Tyr	0.397	0.339	0.397	N/A
61	Oleic acid and Linolenic acid	-0.127	0.237	0.127	N/A
62	Tryptophan	-0.324	0.180	0.324	N/A
63	Glutamine	-0.258	0.137	0.258	N/A
64	Citric acid	-0.184	0.104	0.184	N/A
65	Fru-Asp	0.049	0.065	0.049	N/A
66	Fru-Val	0.175	0.042	0.175	N/A
67	Fru-Thr	0.074	0.022	0.074	N/A
68	Stearic acid	-0.008	0.001	0.008	N/A
69	Fru-Leu	0.012	0.000	0.012	N/A
70	Fru-Ile	-0.003	0.000	0.003	N/A

## 4 Conclusions

This study employed preprocessed data—specifically, morphological features extracted from images and chemical component data predicted from NIR spectra—as inputs to construct multiclass identification models. The proposed combined kernel SVM model demonstrated high accuracy and robustness. In contrast to some previous studies, practical model interpretation was achieved by applying SHAP to the models constructed with these preprocessed data types. The detailed contributions of individual variables were clarified using SHAP summary and dependence plots. Furthermore, the analysis suggested that the absolute value of the slope observed in SHAP dependence plots shows potential as an alternative metric for evaluating variable importance. These results indicate that accurate and robust models can be constructed from imbalanced, preprocessed data using the PSO optimized combined kernel SVM, while simultaneously allowing for practical model interpretation to provide detailed variable analysis. This approach broadens the application scope of image and NIR spectrum data. Looking forward, this methodology is considered transferable and applicable for exploring key variables related to the quality and characteristics of various agricultural products.

## Data Availability

Data and Matlab code are available at github (https://github.com/cvn77andf1/Tobacco-data-and-cod).
