# Supplementary material for: Using preprocessed datasets to construct and interpret multiclass identification models
Source: Front Plant Sci. 2025 Aug 20;16:1597673. doi: 10.3389/fpls.2025.1597673 (PMC12405167; doi:10.3389/fpls.2025.1597673)

**Supporting information**

**Using preprocessed datasets to construct and interpret multiclass identification model**

Cong Wang^1†^, Yufeng Fu^2†^, Ran Wan^1^, Le Zhao^1^, Hongbo Wang^a^, Junwei Guo^1^, Qiang Liu^2^, Shan Li^3^, Shengtao Ma^2^, Zhicai Wang^3^, Wei Huang^3^, Huimin Liu^1^, Song Yang^1*^, Cong Nie^1*^

**Table S1** Accuracy of 10-folds CV of rice dataset using linear SVM

|  | Fold1 | Fold2 | Fold3 | Fold4 | Fold5 | Fold6 | Fold7 | Fold8 | Fold9 | Fold10 | Average |
| --- | --- | --- | --- | --- | --- | --- | --- | --- | --- | --- | --- |
| Basmati | 95.4% | 96.1% | 95.2% | 96.9% | 95.9% | 96.6% | 95.7% | 95.8% | 95.8% | 95.8% | 95.9% |
| Arborio | 96.3% | 96.6% | 96.7% | 96.2% | 95.6% | 96.5% | 96.6% | 96.6% | 95.9% | 96.7% | 96.4% |
| Jasmine | 97.8% | 97.9% | 97.7% | 97.8% | 97.1% | 97.5% | 97.3% | 97.7% | 97.4% | 97.8% | 97.6% |
| Ipsala | 99.1% | 99.4% | 99.1% | 99.5% | 99.1% | 98.9% | 99.2% | 99.2% | 99.1% | 99.1% | 99.2% |
| Karacadag | 98.2% | 97.3% | 96.7% | 96.4% | 98.0% | 97.7% | 97.0% | 97.6% | 97.4% | 96.8% | 97.3% |
| Total | 97.3% | 97.5% | 97.1% | 97.4% | 97.1% | 97.5% | 97.2% | 97.3% | 97.1% | 97.3% | 97.3% |

For section of cultivation regions identification model construction

In the stepwise linear Fisher Discriminant Analysis (FDA), compounds data were standardized before training step. Variable selection was conducted using stepwise method based on Wilks Lambda. No variables screening was conducted before modeling with SVM.

**Table S2** Overall accuracy and average accuracy of 5-folds CV of data set 1

| Method | Linear Fisher | | Linear SVM | |
| --- | --- | --- | --- | --- |
| Accuracy of  Data set 1 | Full data training accuracy | Average accuracy of 5-folds CV | Full data training accuracy | Average accuracy of 5-folds CV |
| Domestic Zone 1 | 95.9% | 94.8% | 99.4% | 98.1% |
| Domestic Zone 2 | 97.3% | 88.0% | 87.8% | 84.0% |
| Domestic Zone 3 | 100.0% | 0.0% | 87.5% | 40.0% |
| Domestic Zone 4 | 98.2% | 98.3% | 98.2% | 94.8% |
| Domestic Zone 5 | 97.8% | 96.4% | 99.3% | 98.6% |
| Domestic Zone 6 | 96.9% | 96.9% | 96.9% | 95.4% |
| Domestic Zone 7 | 100.0% | 0.0% | 100.0% | 100.0% |
| Domestic Zone 8 | 100.0% | 0.0% | 85.7% | 90.0% |
| Brazil | 97.9% | 93.8% | 97.9% | 94.0% |
| Zimbabwe | 100.0% | 100.0% | 100.0% | 96.7% |
| Total Accuracy | 97.0% | 92.3% | 97.7% | 95.4% |

**Table S3** 5-folds CV accuracy of tobacco dataset 1 using Linear FDA

|  | Fold 1 | Fold 2 | Fold 3 | Fold 4 | Fold 5 | Average |
| --- | --- | --- | --- | --- | --- | --- |
| Domestic Zone 1 | 95.9% | 91.8% | 95.9% | 94.4% | 95.8% | 94.8% |
| Domestic Zone 2 | 80.0% | 73.3% | 100.0% | 93.3% | 93.3% | 88.0% |
| Domestic Zone 3 | 0.0% | 0.0% | 0.0% | 0.0% | 0.0% | 0.0% |
| Domestic Zone 4 | 91.7% | 100.0% | 100.0% | 100.0% | 100.0% | 98.3% |
| Domestic Zone 5 | 100.0% | 100.0% | 89.3% | 96.4% | 96.4% | 96.4% |
| Domestic Zone 6 | 100.0% | 100.0% | 84.6% | 100.0% | 100.0% | 96.9% |
| Domestic Zone 7 | 0.0% | 0.0% | 0.0% | 0.0% | 0.0% | 0.0% |
| Domestic Zone 8 | 0.0% | 0.0% | 0.0% | 0.0% | 0.0% | 0.0% |
| Brazil | 88.9% | 100.0% | 80.0% | 100.0% | 100.0% | 93.8% |
| Zimbabwe | 100.0% | 100.0% | 100.0% | 100.0% | 100.0% | 100.0% |
| Total Right % | 91.9% | 90.6% | 91.2% | 93.7% | 94.3% | 92.3% |

**Table S4** 5-folds CV accuracy of tobacco dataset 1 using Linear SVM

|  | Fold 1 | Fold 2 | Fold 3 | Fold 4 | Fold 5 | Average |
| --- | --- | --- | --- | --- | --- | --- |
| Domestic Zone 1 | 100.0% | 95.9% | 98.6% | 98.6% | 97.2% | 98.1% |
| Domestic Zone 2 | 80.0% | 66.7% | 100.0% | 93.3% | 80.0% | 84.0% |
| Domestic Zone 3 | 100.0% | 0.0% | 50.0% | 0.0% | 50.0% | 40.0% |
| Domestic Zone 4 | 83.3% | 90.9% | 100.0% | 100.0% | 100.0% | 94.8% |
| Domestic Zone 5 | 100.0% | 100.0% | 92.9% | 100.0% | 100.0% | 98.6% |
| Domestic Zone 6 | 92.3% | 100.0% | 84.6% | 100.0% | 100.0% | 95.4% |
| Domestic Zone 7 | 100.0% | 100.0% | 100.0% | 100.0% | 100.0% | 100.0% |
| Domestic Zone 8 | 50.0% | 100.0% | 100.0% | 100.0% | 100.0% | 90.0% |
| Brazil | 100.0% | 100.0% | 80.0% | 90.0% | 100.0% | 94.0% |
| Zimbabwe | 100.0% | 100.0% | 100.0% | 83.3% | 100.0% | 96.7% |
| Total Right % | 95.6% | 93.8% | 95.0% | 96.2% | 96.2% | 95.4% |

**Table S5** 5-folds CV accuracy of tobacco dataset 1 using Polynomial kernel SVM, Polynomial parameters: a=1/65, b=1.5, q=3

|  | Fold 1 | Fold 2 | Fold 3 | Fold 4 | Fold 5 | Average |
| --- | --- | --- | --- | --- | --- | --- |
| Domestic Zone 1 | 100.0% | 97.3% | 100.0% | 100.0% | 98.6% | 99.2% |
| Domestic Zone 2 | 86.7% | 73.3% | 100.0% | 86.7% | 86.7% | 86.7% |
| Domestic Zone 3 | 100.0% | 100.0% | 50.0% | 100.0% | 50.0% | 80.0% |
| Domestic Zone 4 | 91.7% | 100.0% | 100.0% | 100.0% | 100.0% | 98.3% |
| Domestic Zone 5 | 100.0% | 100.0% | 96.4% | 100.0% | 96.4% | 98.6% |
| Domestic Zone 6 | 92.3% | 100.0% | 84.6% | 92.3% | 100.0% | 93.8% |
| Domestic Zone 7 | 100.0% | 100.0% | 100.0% | 100.0% | 100.0% | 100.0% |
| Domestic Zone 8 | 50.0% | 100.0% | 100.0% | 100.0% | 100.0% | 90.0% |
| Brazil | 100.0% | 100.0% | 90.0% | 90.0% | 100.0% | 96.0% |
| Zimbabwe | 100.0% | 100.0% | 100.0% | 100.0% | 100.0% | 100.0% |
| Right percentage | 96.9% | 96.3% | 96.9% | 97.5% | 96.9% | 96.9% |

**Table S6** 5-folds CV accuracy of tobacco dataset 1 using RBF SVM, kernel parameters: sigma^2=32

|  | Fold 1 | Fold 2 | Fold 3 | Fold 4 | Fold 5 | Average |
| --- | --- | --- | --- | --- | --- | --- |
| Domestic Zone 1 | 100.0% | 97.3% | 100.0% | 100.0% | 100.0% | 99.5% |
| Domestic Zone 2 | 66.7% | 60.0% | 85.7% | 93.3% | 60.0% | 73.1% |
| Domestic Zone 3 | 0.0% | 100.0% | 50.0% | 50.0% | 50.0% | 50.0% |
| Domestic Zone 4 | 83.3% | 100.0% | 100.0% | 100.0% | 100.0% | 96.7% |
| Domestic Zone 5 | 100.0% | 96.4% | 92.9% | 100.0% | 100.0% | 97.9% |
| Domestic Zone 6 | 92.3% | 100.0% | 84.6% | 92.3% | 100.0% | 93.8% |
| Domestic Zone 7 | 100.0% | 100.0% | 100.0% | 100.0% | 100.0% | 100.0% |
| Domestic Zone 8 | 0.0% | 100.0% | 50.0% | 100.0% | 100.0% | 70.0% |
| Brazil | 80.0% | 100.0% | 100.0% | 100.0% | 80.0% | 92.0% |
| Zimbabwe | 100.0% | 100.0% | 100.0% | 100.0% | 100.0% | 100.0% |
| Right percentage | 92.5% | 94.4% | 95.0% | 98.1% | 94.3% | 94.9% |

**Table S7** 5-folds CV accuracy of tobacco dataset 1 using sigmoid kernel SVM, kernel parameters: a=1/65,b=-1.2

|  | Fold 1 | Fold 2 | Fold 3 | Fold 4 | Fold 5 | Average |
| --- | --- | --- | --- | --- | --- | --- |
| Domestic Zone 1 | 100.0% | 98.6% | 98.6% | 98.6% | 100.0% | 99.2% |
| Domestic Zone 2 | 20.0% | 33.3% | 35.7% | 13.3% | 26.7% | 25.8% |
| Domestic Zone 3 | 0.0% | 0.0% | 0.0% | 0.0% | 0.0% | 0.0% |
| Domestic Zone 4 | 91.7% | 90.9% | 100.0% | 100.0% | 100.0% | 96.5% |
| Domestic Zone 5 | 100.0% | 92.9% | 89.3% | 92.9% | 100.0% | 95.0% |
| Domestic Zone 6 | 84.6% | 92.3% | 84.6% | 92.3% | 100.0% | 90.8% |
| Domestic Zone 7 | 100.0% | 100.0% | 100.0% | 0.0% | 100.0% | 80.0% |
| Domestic Zone 8 | 100.0% | 0.0% | 50.0% | 100.0% | 100.0% | 70.0% |
| Brazil | 90.0% | 100.0% | 100.0% | 100.0% | 90.0% | 96.0% |
| Zimbabwe | 100.0% | 100.0% | 100.0% | 100.0% | 100.0% | 100.0% |
| Right percentage | 89.4% | 89.4% | 88.7% | 87.4% | 91.2% | 89.2% |

**Table S8** Confusion matrix of tobacco dataset 1, model constructed on full dataset, Kernel parameters were listed in Table S10.

| Predict  Real | Domestic Zone 1 | Domestic Zone 2 | Domestic Zone 3 | Domestic Zone 4 | Domestic Zone 5 | Domestic Zone 6 | Domestic Zone 7 | Domestic Zone 8 | Brazil | Zimbabwe | Amount of sample | Right percentage | F1-score |
| --- | --- | --- | --- | --- | --- | --- | --- | --- | --- | --- | --- | --- | --- |
| Domestic Zone 1 | 363 | 0 | 0 | 0 | 0 | 0 | 0 | 0 | 0 | 0 | 363 | 100.0% | 0.995 |
| Domestic Zone 2 | 2 | 71 | 0 | 0 | 1 | 0 | 0 | 0 | 0 | 0 | 74 | 95.9% | 0.979 |
| Domestic Zone 3 | 0 | 0 | 7 | 1 | 0 | 0 | 0 | 0 | 0 | 0 | 8 | 87.5% | 0.933 |
| Domestic Zone 4 | 1 | 0 | 0 | 55 | 0 | 0 | 0 | 0 | 0 | 0 | 56 | 98.2% | 0.982 |
| Domestic Zone 5 | 0 | 0 | 0 | 0 | 138 | 1 | 0 | 0 | 0 | 0 | 139 | 99.3% | 0.989 |
| Domestic Zone 6 | 1 | 0 | 0 | 0 | 1 | 63 | 0 | 0 | 0 | 0 | 65 | 96.9% | 0.969 |
| Domestic Zone 7 | 0 | 0 | 0 | 0 | 0 | 0 | 7 | 0 | 0 | 0 | 7 | 100.0% | 1.00 |
| Domestic Zone 8 | 0 | 0 | 0 | 0 | 0 | 0 | 0 | 6 | 0 | 1 | 7 | 85.7% | 0.923 |
| Brazil | 0 | 0 | 0 | 0 | 0 | 1 | 0 | 0 | 47 | 0 | 48 | 97.9% | 0.989 |
| Zimbabwe | 0 | 0 | 0 | 0 | 0 | 0 | 0 | 0 | 0 | 30 | 30 | 100.0% | 0.984 |
| Precision | 98.9% | 100.0% | 100.0% | 98.2% | 98.6% | 96.9% | 100.0% | 100.0% | 100.0% | 96.8% | Total Accuracy | 98.7% |  |

**Table S9** 5-folds CV form of tobacco dataset 1 using combined kernel function, Kernel parameters were listed in Table S10.

|  | Fold 1 | Fold 2 | Fold 3 | Fold 4 | Fold 5 | Average |
| --- | --- | --- | --- | --- | --- | --- |
| Domestic Zone 1 | 100.0% | 98.6% | 100.0% | 100.0% | 100.0% | 99.7% |
| Domestic Zone 2 | 86.7% | 80.0% | 100.0% | 86.7% | 86.7% | 88.0% |
| Domestic Zone 3 | 100.0% | 100.0% | 50.0% | 100.0% | 50.0% | 80.0% |
| Domestic Zone 4 | 91.7% | 100.0% | 100.0% | 100.0% | 100.0% | 98.3% |
| Domestic Zone 5 | 100.0% | 100.0% | 96.4% | 100.0% | 96.4% | 98.6% |
| Domestic Zone 6 | 100.0% | 100.0% | 84.6% | 92.3% | 100.0% | 95.4% |
| Domestic Zone 7 | 100.0% | 100.0% | 100.0% | 100.0% | 100.0% | 100.0% |
| Domestic Zone 8 | 50.0% | 100.0% | 100.0% | 100.0% | 100.0% | 90.0% |
| Brazil | 100.0% | 100.0% | 90.0% | 90.0% | 100.0% | 96.0% |
| Zimbabwe | 100.0% | 100.0% | 100.0% | 100.0% | 100.0% | 100.0% |
| Total Accuracy | 97.5% | 97.5% | 96.9% | 97.5% | 97.5% | 97.4% |

**Table S10** Kernel parameters optimized by PSO (for tobacco dataset 1)

|  | Kernel percentage | | | Kernel parameters | | | |
| --- | --- | --- | --- | --- | --- | --- | --- |
|  |  |  |  | Polynomial | | | RBF |
|  | linear | polynomial | RBF | a | b | q | $\sigma^{2}$ |
| data set 1 | 0% | 44.5% | 55.5% | 65 | 1.5 | 3 | 32 |

Over-sampling

Sample from class with small amount was picked and random value within ±3% (empirical percent from our analytical chemistry experiments) of original value was added to avoid exactly same data. From the Table below, over-sample made a plausible improvement in Fisher discrimination. The average recall of zone 7 and 8 got improved from 0% to 90%. However, over-sampling only achieved very limited improvement from 0% to 10%. In the SVM method, the result of over-sample is unsatisfied. The only improvement was achieved in zone 3 among zone 3, 7 and 8 with linear kernel. With Polynomial kernel, the recall of zone 3 got lower. Meanwhile, the recall of same other zone, including zone 1, 4 and Zimbabwe, got lower. The total accuracy also decreased. Compared to the other method, the hybrid kernel SVM achieved highest recall in most zone and highest total accuracy.

**Table S11** Recall/Accuracy table of 5-fold CV with/without Over-sampling

| Method | Fisher | | SVM | | | | |
| --- | --- | --- | --- | --- | --- | --- | --- |
| Parameters | / | / | linear | linear | Polynomial | Polynomial | SAKSVM |
| Over-sampling | yes | no | yes | no | yes | no | no |
| Domestic Zone 1 | 92.8% | 94.8% | 93.9% | 98.1% | 98.6% | 99.2% | 99.7% |
| Domestic Zone 2 | 86.7% | 88.0% | 86.6% | 84.0% | 85.3% | 86.7% | 88.0% |
| Domestic Zone 3 | 10.0% | 0.0% | 80.0% | 40.0% | 70.0% | 80.0% | 80.0% |
| Domestic Zone 4 | 98.3% | 98.3% | 91.2% | 94.8% | 89.2% | 98.3% | 98.3% |
| Domestic Zone 5 | 96.4% | 96.4% | 97.1% | 98.6% | 98.6% | 98.6% | 98.6% |
| Domestic Zone 6 | 96.9% | 96.9% | 96.9% | 95.4% | 93.8% | 93.8% | 95.4% |
| Domestic Zone 7 | 90.0% | 0.0% | 100.0% | 100.0% | 100.0% | 100.0% | 100.0% |
| Domestic Zone 8 | 90.0% | 0.0% | 90.0% | 90.0% | 90.0% | 90.0% | 90.0% |
| Brazil | 98.0% | 93.8% | 96.0% | 94.0% | 96.0% | 96.0% | 96.0% |
| Zimbabwe | 96.7% | 100.0% | 93.3% | 96.7% | 93.3% | 100.0% | 100.0% |
| Total accuracy | 93.1% | 92.3% | 93.7% | 95.4% | 95.5% | 96.9% | 97.4% |

**Table S12** t-value of paired samples t-test, two-tailed test, 95% CI, t_(0.975,4)_=2.7764

|  |  | Method | Fisher | | SVM | | | | |
| --- | --- | --- | --- | --- | --- | --- | --- | --- | --- |
|  |  | Parameters | / | / | linear | linear | Polynomial | Polynomial | hybrid kernel |
| Method | Parameters | over-sampling | yes | no | yes | no | yes | no | no |
| Fisher | / | yes | --- | --- | --- | --- | --- | --- | --- |
|  | / | no | 0.9666 | --- | --- | --- | --- | --- | --- |
| SVM | linear | yes | 0.5970 | 2.7574 | --- | --- | --- | --- | --- |
|  | linear | no | 3.3523 | 8.2700 | 3.8250 | --- | --- | --- | --- |
|  | Polynomial | yes | 4.4395 | 4.2243 | 2.2526 | 0.2998 | --- | --- | --- |
|  | Polynomial | no | 7.8126 | 7.4599 | 4.7661 | 4.7238 | 5.8891 | --- | --- |
|  | hybrid kernel | no | 7.5920 | 7.3982 | 5.4661 | 4.4435 | 4.3512 | 2.2632 | --- |

**Table S13** Model evaluation of tobacco dataset 2

| Predicting Accuracy | 5-folds CV ( on training set) | | | | | | training set | Independent test set |
| --- | --- | --- | --- | --- | --- | --- | --- | --- |
|  | fold 1 | fold 2 | fold 3 | fold 4 | fold 5 | Average |  |  |
| Domestic Zone 1 | 100.0% | 100.0% | 92.9% | 92.9% | 100.0% | 97.1% | 100.0% | 100.0% |
| Domestic Zone 2 | 85.7% | 85.7% | 83.3% | 83.3% | 83.3% | 84.3% | 100.0% | 87.5% |
| Domestic Zone 3 | 100.0% | 100.0% | 100.0% | 100.0% | 100.0% | 100.0% | 100.0% | 80.0% |
| Domestic Zone 4 | 96.2% | 96.2% | 100.0% | 100.0% | 100.0% | 98.5% | 99.2% | 97.0% |
| Domestic Zone 5 | 100.0% | 100.0% | 100.0% | 66.7% | 33.3% | 80.0% | 100.0% | 100.0% |
| Domestic Zone 6 | 100.0% | 100.0% | 100.0% | 100.0% | 100.0% | 100.0% | 100.0% | 100.0% |
| Domestic Zone 8 | 100.0% | 100.0% | 100.0% | 75.0% | 100.0% | 95.0% | 100.0% | 100.0% |
| Brazil | 100.0% | 100.0% | 100.0% | 100.0% | 91.7% | 98.3% | 100.0% | 100.0% |
| Zimbabwe | 100.0% | 91.7% | 90.0% | 100.0% | 100.0% | 96.3% | 100.0% | 100.0% |
| America | 100.0% | 100.0% | 100.0% | 100.0% | 100.0% | 100.0% | 100.0% | 100.0% |
| Zambia | 100.0% | 50.0% | 100.0% | 50.0% | 50.0% | 70.0% | 100.0% | 100.0% |
| Total accuracy | 98.0% | 96.1% | 96.1% | 94.1% | 95.1% | 95.9% | 99.8% | 97.7% |

**Table S14** Confusion matrix of independent test set of tobacco dataset 2, kernel parameters were listed in Table S14.

| Predict  Real | Domestic Zone 1 | Domestic Zone 2 | Domestic Zone 3 | Domestic Zone 4 | Domestic Zone 5 | Domestic Zone 6 | Domestic Zone 8 | Brazil | Zimbabwe | America | Zambia | Amount of sample | Right percentage | F1-Score |
| --- | --- | --- | --- | --- | --- | --- | --- | --- | --- | --- | --- | --- | --- | --- |
| Domestic Zone 1 | 35 | 0 | 0 | 0 | 0 | 0 | 0 | 0 | 0 | 0 | 0 | 35 | 100.0% | 0.958 |
| Domestic Zone 2 | 1 | 7 | 0 | 0 | 0 | 0 | 0 | 0 | 0 | 0 | 0 | 8 | 87.5% | 0.933 |
| Domestic Zone 3 | 1 | 0 | 4 | 0 | 0 | 0 | 0 | 0 | 0 | 0 | 0 | 5 | 80.0% | 0.888 |
| Domestic Zone 4 | 1 | 0 | 0 | 32 | 0 | 0 | 0 | 0 | 0 | 0 | 0 | 33 | 97.0% | 0.984 |
| Domestic Zone 5 | 0 | 0 | 0 | 0 | 3 | 0 | 0 | 0 | 0 | 0 | 0 | 3 | 100.0% | 1 |
| Domestic Zone 6 | 0 | 0 | 0 | 0 | 0 | 7 | 0 | 0 | 0 | 0 | 0 | 7 | 100.0% | 1 |
| Domestic Zone 8 | 0 | 0 | 0 | 0 | 0 | 0 | 4 | 0 | 0 | 0 | 0 | 4 | 100.0% | 1 |
| Brazil | 0 | 0 | 0 | 0 | 0 | 0 | 0 | 15 | 0 | 0 | 0 | 15 | 100.0% | 1 |
| Zimbabwe | 0 | 0 | 0 | 0 | 0 | 0 | 0 | 0 | 12 | 0 | 0 | 12 | 100.0% | 1 |
| America | 0 | 0 | 0 | 0 | 0 | 0 | 0 | 0 | 0 | 4 | 0 | 4 | 100.0% | 1 |
| Zambia | 0 | 0 | 0 | 0 | 0 | 0 | 0 | 0 | 0 | 0 | 2 | 2 | 100.0% | 1 |
| Precision | 92.1% | 100.0% | 100.0% | 100.0% | 100.0% | 100.0% | 100.0% | 100.0% | 100.0% | 100.0% | 100.0% | Total Right percentage | 97.7% |  |

**Table S15** Kernel parameters optimized by PSO (for tobacco dataset 2)

|  | Kernel percentage | | | Kernel parameters | | | |
| --- | --- | --- | --- | --- | --- | --- | --- |
|  |  |  |  | Polynomial | | | RBF |
|  | linear | polynomial | RBF | a | b | q | $\sigma^{2}$ |
| data set 1 | 33.3% | 25.0% | 41.7% | 47 | 2.2 | 2 | 18 |

**Table S16** Confusion matrix external validation of model (constructed on tobacco dataset 2, No American sample in testset)

| Predict  Real | Domestic Zone 1 | Domestic Zone 2 | Domestic Zone 3 | Domestic Zone 4 | Domestic Zone 5 | Domestic Zone 6 | Domestic Zone 8 | Brazil | Zimbabwe | America | Zambia | Amount of sample | Right percentage | F1-Score |
| --- | --- | --- | --- | --- | --- | --- | --- | --- | --- | --- | --- | --- | --- | --- |
| Domestic Zone 1 | 325 | 8 | 0 | 0 | 0 | 0 | 0 | 0 | 0 | 0 | 0 | 333 | 97.6% | 0.977444 |
| Domestic Zone 2 | 4 | 25 | 0 | 0 | 0 | 0 | 0 | 0 | 0 | 0 | 0 | 29 | 86.2% | 0.632911 |
| Domestic Zone 3 | 1 | 4 | 6 | 1 | 0 | 0 | 0 | 0 | 0 | 0 | 0 | 12 | 50.0% | 0.666667 |
| Domestic Zone 4 | 1 | 0 | 0 | 262 | 0 | 0 | 0 | 0 | 1 | 0 | 0 | 264 | 99.2% | 0.994307 |
| Domestic Zone 5 | 0 | 6 | 0 | 0 | 5 | 0 | 0 | 0 | 0 | 0 | 0 | 11 | 45.5% | 0.588235 |
| Domestic Zone 6 | 1 | 0 | 0 | 0 | 1 | 27 | 0 | 1 | 0 | 0 | 0 | 30 | 90.0% | 0.947368 |
| Domestic Zone 8 | 0 | 0 | 0 | 0 | 0 | 0 | 39 | 0 | 0 | 0 | 0 | 39 | 100.0% | 1 |
| Brazil | 0 | 1 | 0 | 0 | 0 | 0 | 0 | 42 | 0 | 0 | 0 | 43 | 97.7% | 1 |
| Zimbabwe | 0 | 3 | 0 | 0 | 0 | 0 | 0 | 0 | 45 | 0 | 0 | 48 | 93.8% | 1 |
| America | 0 | 0 | 0 | 0 | 0 | 0 | 0 | 0 | 0 | 0 | 0 | 0 | NaN | NaN |
| Zambia | 0 | 3 | 0 | 0 | 0 | 0 | 0 | 0 | 4 | 0 | 20 | 27 | 74.1% | 1 |
| Precision | 97.9% | 50.0% | 100.0% | 99.6% | 83.3% | 100.0% | 100.0% | 97.7% | 90.0% | NaN | 100.0% | Total Right percentage | 95.2% |  |

**Fig. S1.** Heatmap of 70 compounds of tobacco dataset 1


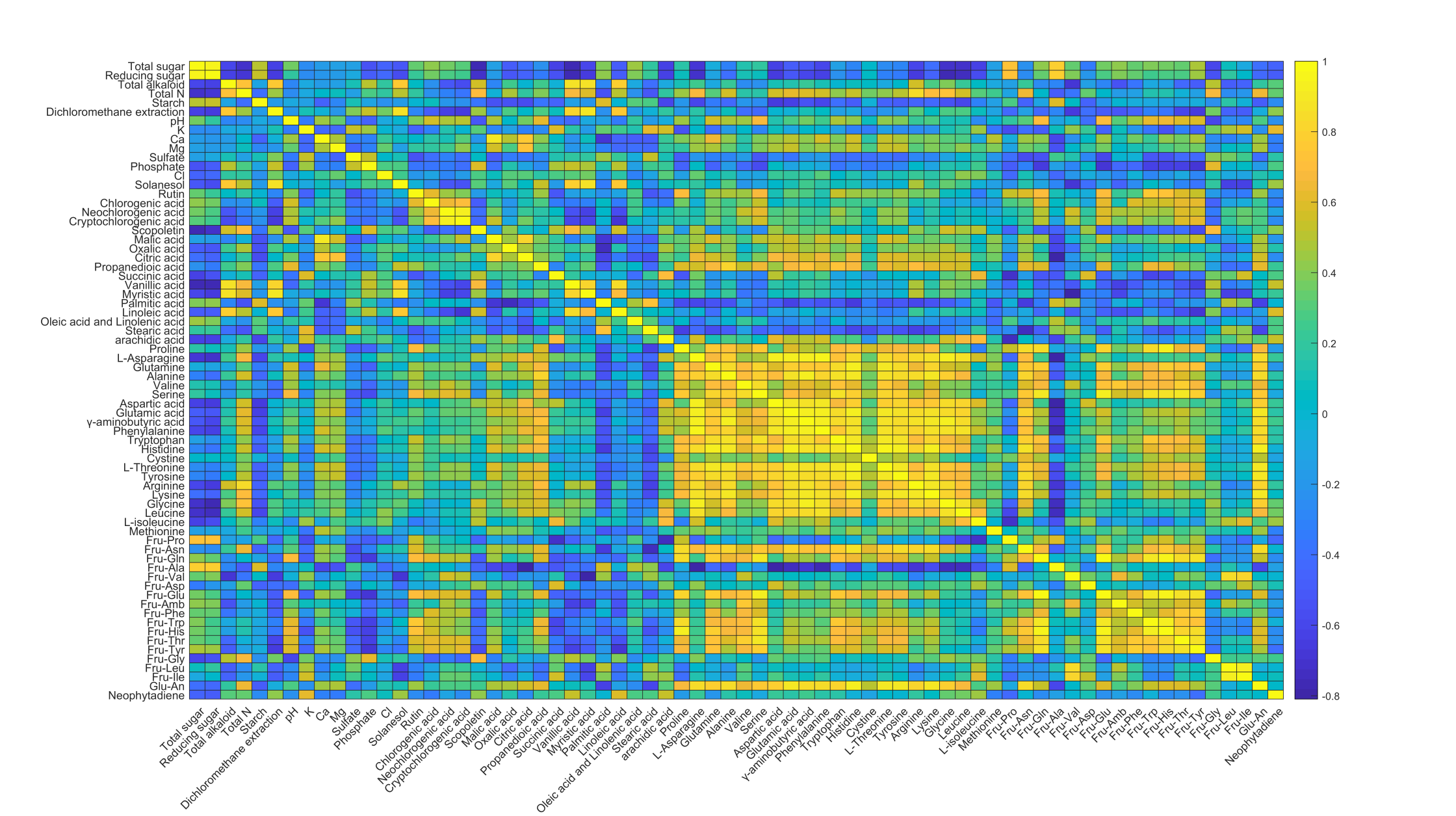

Supplement: Supplementary file 1 [file DataSheet1.docx]
